# Quantitative Assessment of Parkinsonian Tremor Based on an Inertial Measurement Unit

**DOI:** 10.3390/s151025055

**Published:** 2015-09-29

**Authors:** Houde Dai, Pengyue Zhang, Tim C. Lueth

**Affiliations:** 1 Quanzhou Institute of Equipment Manufacturing, Haixi Institutes, Chinese Academy of Sciences, Bolan Av., Jinjiang 362200, China; 2 Institute of Micro Technology and Medical Device Technology, Technische Universitaet Muenchen, 85748 Garching, Germany; E-Mail: tim.lueth@tum.de; 3 Medical Faculty, Kunming University of Science and Technology, Kunming 650500, China; E-Mail: zpy19802000@163.com

**Keywords:** Inertial measurement unit, wearable system, tremor quantification, Parkinson’s disease, technical validation

## Abstract

Quantitative assessment of parkinsonian tremor based on inertial sensors can provide reliable feedback on the effect of medication. In this regard, the features of parkinsonian tremor and its unique properties such as motor fluctuations and dyskinesia are taken into account. Least-square-estimation models are used to assess the severities of rest, postural, and action tremors. In addition, a time-frequency signal analysis algorithm for tremor state detection was also included in the tremor assessment method. This inertial sensor-based method was verified through comparison with an electromagnetic motion tracking system. Seven Parkinson’s disease (PD) patients were tested using this tremor assessment system. The measured tremor amplitudes correlated well with the judgments of a neurologist (*r* = 0.98). The systematic analysis of sensor-based tremor quantification and the corresponding experiments could be of great help in monitoring the severity of parkinsonian tremor.

## 1. Introduction

It is estimated that 1% of 70-year-olds suffer from Parkinson’s disease (PD) [1]. At present, about four to six million people are PD patients, in which about 10% of PD patients are younger than 50 years old [2]. Due to demographic increase of the elderly population, PD will occur more frequently in the future.

Parkinsonian tremor (oscillatory movement) is the central symptom of PD, presenting in about 70% of PD cases [3]. It can be judged in the patient’s hands, feet, and head with a particular frequency (3.5–7.5 Hz) and amplitude (speed and range) [4]. Tremors often begin from one finger and expand to the whole arm, with the rhythmic back-and-forth action called the “pill-rolling” tremor [5]. The “pill-rolling” tremor takes its name from the action of rolling a pill with the thumb and index finger.

Parkinsonian tremor manifests in different types: rest, postural, and action tremors. Rest tremor, which is the characteristic of parkinsonian tremor, happens when a body part is relaxed. The postural tremor occurs while a body part is held straight out from the body in a stable position against gravity. The action tremor (kinetic tremor) happens when a voluntary contraction of a muscle follows a certain action—for example, holding a cup [6]. Pure rest tremor is rare. For a PD patient, postural or action tremors appear together with rest tremor, but with different frequencies. A rest tremor may be combined in a postural tremor, but disappears during an action tremor task. An action tremor occurs in most PD; however, the tremor severity is not associated with age or disease duration [7].

The precise mechanism of PD remains uncertain at present [8]. The Unified Parkinson’s Disease Rating Scale (UPDRS), which is a subjective assessment performed by the qualitative judgment of neurologists, is the clinical standard for parkinsonian tremor assessment. UPDRS tremor ratings are from 0 to 4, which denote normal, slight, mild, moderate, and severe, respectively.

Spiral drawing tests by a parkinsonian patient are used to judge the severity of parkinsonian tremor [7]. However, there is no practical instrumental method for the accurate monitoring of the PD symptoms as of yet. Nevertheless, doctors and patients are eager for objective severity assessment of parkinsonian tremor [9].

In addition, about 50% of PD patients have side effects such as dyskinesia and motor fluctuations after five years of levodopa medication. The occurrence and amplitude of parkinsonian tremor are influenced by such effects [10,11].

Timmer *et al.* reported that parkinsonian tremor exhibits a two-order nonlinear and no-strictly periodic oscillation [12]. Because the dominant frequency of parkinsonian tremor has no direct relationship with tremor severity, the tremor severity can be rated only by tremor range (*i.e.*, amplitude) [13].

To quantify neurological symptoms during deep-brain surgery, we have presented a wearable system for tremor quantitative assessment [14,15,16,17].

The goal of this study is to investigate the features of parkinsonian tremor and to develop a sensor-based quantitative assessment method. In this concept, the current possibilities of inertial sensor technology and motion-tracking algorithms can be used to implement quantitative assessments of parkinsonian tremor.

## 2. State of the Art and Task Description

### 2.1. State of the Art in Parkinsonian Tremor Quantification

Some research groups have used infrared cameras, video tracking, digital drawing tablets, laser-based displacement transducers, and electromyography (EMG) to assess tremors objectively [1,2,3,4,5,6,7,8,9,10]. Sensors were placed on the body, feet or arms of the patient. Most of these sensor-based systems could detect the change between therapy “OFF” and “ON” states during medication. However, because these systems have big dimensions or patients were uncomfortable wearing such devices, such systems have limited usability in clinical applications [7].

At present, most tremor assessment methods were based on inertial sensors and a computer-based system. There has recently been growing interest in the application of MEMS (Micro-Electro-Mechanical Systems) inertial sensors for continuous monitoring of PD symptoms. With the development of new MEMS technology, the dimension of the sensor circuit board is smaller and signal processing is easier to carry out than before [5].

Some studies have indicated that sensor data from accelerometers and gyroscopes correlate strongly with UPDRS tremor ratings [5,18,19,20,21,22].

The research carried out by Elble *et al.*, which enrolled 928 patients, indicated that tremor amplitude is logarithmically correlated to the five-point UPDRS scale [20].

The Kinesia™ system (Great Lakes NeuroTechnologies Inc., Cleveland, OH, USA), which has been approved by the FDA (the United States Food and Drug Administration) for sale, is used to assess parkinsonian symptoms with an inertial measurement unit (IMU), which embeds a three-axis gyroscope and a three-axis accelerometer in a single chip, on the top side of a finger [20]. Giuffrida’s research indicated that the logarithm of the peak powers’ summation of power spectrums of both accelerometer and gyroscope signals, during rest and postural tremor tasks, correlated best with UPDRS tremor scale (the coefficient of determination *r*^2^ = 0.9) [23]. At the same time, the RMS summation of both accelerometer and gyroscope signals, during action tremor tasks, correlated best with UPDRS tremor ratings (*r*^2^ = 0.69) [21].

In addition, Niazmand *et al.* presented a glove system to quantitatively assess parkinsonian tremor based on accelerometers [18]. Salarian *et al.* presented an ambulatory tremor monitor with a three-axis gyroscope on the wrist [4]. The Motus Movement Monitor (Motus Bioengineering Inc., Benicia, CA, USA), based on a three-axis gyroscope on the top side of the patient’s palm, is used to assess tremor and other symptoms [24]. Pierleoni *et al.* used a wrist module, consists of an accelerometer and a gyroscope, to classify and qualitatively assess tremors [25]. Khan *et al.* presented a wearable accelerometer system to classify tremors based on several algorithms, especially the non-linear radial basis function kernel [26]. Zhang *et al.* developed an accelerometer sensing system that adopted the short-time Fourier transform to reduce the instability of the tremor signals [27].

Beginning in August 2014, the Michael J. Fox Foundation for Parkinson’s Research (MJFF) and Intel Corporation have joined forces to improve Parkinson’s disease monitoring via big data analytics and data from wearable computing. Open-access sensor data (accelerometer, compass, *etc.*) are available now [28].

### 2.2. Special Concerns for Side Effects and Task Description

Motor fluctuations, without being considered by other tremor assessment systems, indicate that the effective period of certain doses is shorter all the time, with the name of end-of-dose deterioration. Motor fluctuations also represent the alternations between “ON” (a state of good response to anti-parkinsonian medications) and “OFF” (a state for patients experiencing parkinsonian symptoms). The symptoms of a PD patient may reappear unexpectedly and suddenly, a switch sensation described as “ON/OFF” syndrome. Levodopa-induced dyskinesia (LID) involves a series of hyperkinetic movements (involuntary, episodic, and irregular) such as athetosis, chorea, and dystonia [10]. The occurrence and severity of parkinsonian tremor are influenced by such side effects. Therefore, special concerns for MD and LID should be adopted in the tremor assessment.

The research conducted by Burkhard *et al.* shows that the RMS of the power spectrum of hand-attached gyroscope signals, from 0.25 to 3.25 Hz, during dyskinesia task correlated well with the five-point clinical ratings of dyskinesia severity [24]. The influence of dyskinesia during a tremor task can be removed by applying a high-pass filter, with the cutoff frequency (*f_c_*) set at 3.25 Hz, to the measured IMU signals.

For a PD patient, only the tremor amplitude is required, because the specific tremor frequency does not change for a certain period. In this study, the logarithm of the peak powers’ summation of the IMU sensor signals is regarded as the tremor amplitude. The tremor amplitudes, which are regressed from the peak powers of the IMU signals, can be gauged as the clinician ratings [29].

## 3. Quantitative Assessment of Parkinsonian Tremor

### 3.1. Test Tasks and Relevant Parameters

**Figure 1:**
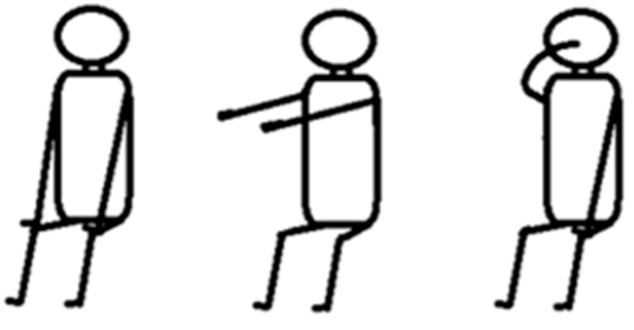
Test tasks for parkinsonian tremor assessment: (**left action**) rest tremor, (**middle action**) postural tremor, and (**right action**) action tremor.

According to the suggestions of neurologists, several tasks are chosen to assess the symptom severities of parkinsonian tremor. As shown in Figure 1, tremor assessment includes three tasks: rest tremor, postural tremor, and action tremor. Each assessment task lasts ten seconds.

The parameters that should be extracted from sensor signal processing and displayed in the graphical user interface (GUI) are:

1. amplitude of parkinsonian tremor (*R*); and
2. dominant frequency of parkinsonian tremor (*F*).

The dominant frequency of parkinsonian tremor is only used as a reference parameter to judge whether it is the range of parkinsonian tremor (3.5–7.5 Hz).

### 3.2. System Concept

**Figure 2:**
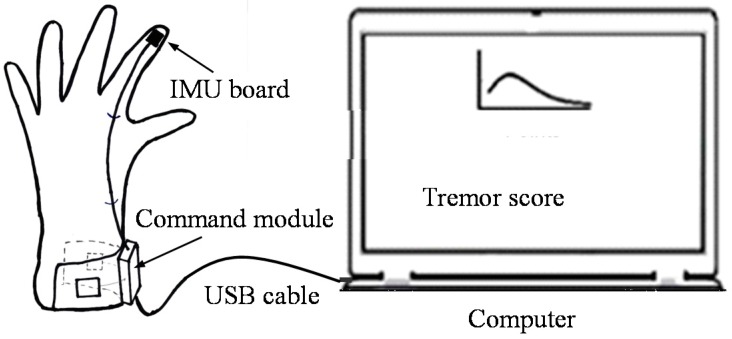
System diagram of the tremor assessment system. This system consists of two parts: glove part (**left**) and computer part (**right**).

A portable parkinsonian tremor assessment system was used in this study [14,15,16,17]. Its system diagram is shown in Figure 2. The reason why use wired communication is for radio safety in the operating room. For home application, a wireless communication interface can be adopted.

A six-axis IMU (MPU6050, InvenSense Inc., San Jose, CA, USA) is placed on the top of the index finger for tremor assessment. MPU6050 has very small dimensions (4 mm × 4 mm × 0.9 mm). A textile glove, incorporating a command module and an IMU circuit board, was placed on the top of middle index finger. The command module sent the sensor data to the computer via a wired USB cable.

The sampling rate of the tremor assessment system was working at 100 Hz. The sensor data from the command module are sent to the computer via a serial-to-USB transmission [30].

### 3.3. Parkinsonian Tremor Assessment Methods

The algorithm used to quantify the severity of parkinsonian tremor is very important. Tremor Quantification has been performed by signal processing methods such as time-domain analysis, time-frequency analysis, spectral analysis, and nonlinear analysis [31,32]. Currently, spectral analysis is used for the majority of these studies [33,34].

In this study, signal processing involves FIR (finite impulse response) filters and IIR (infinite impulse response) filters as well as other special algorithms such as power spectral density (PSD) analysis. To detect tremors, the signals are then processed with PSD calculation (auto power spectrum) with a certain time interval (3–10 s).

For range (displacement) analysis, the angular velocity obtained from the gyroscope needs to be integrated over time only once, but the integration of the acceleration signals requires double integration. However, the sensor bias and drifts are integrated as well. Thus, the IMU raw data were used for the tremor quantification.

The three-axis accelerometer outputs include gravitational acceleration, which equals 9.81 m/s^2^ in vector product, and linear acceleration. Gravitational acceleration can be removed from the accelerometer outputs with high-pass filters. Thus, there are only linear acceleration data for the following signal processing.

Quadratic mean (RMS) interprets actual vibration levels, while PSD results show dominant frequency that contribute the most to the tremor. Because the tremors are based on a dominant frequency, the advantages of PSD compared to a statistical measure (quadratic mean) are that it highlights the tremor signals from noise and other movement with analysis in the frequency dimension and the squared value of the signals.

### 3.4. PSD Analysis for Tremor Signal

Given its oscillatory nature, tremors quantification is suitable to the spectral analysis (PSD method) [34].

The dominant frequency can be calculated using PSD estimation. If the dominant frequencies in different axes are not the same, the valid dominant frequency in the axis with the highest peak power is defined as the dominant frequency of all axes.

Heldman *et al.* presented the discovery that the logarithm of the peak power in all triple-axis accelerations and three-axis angular velocities correlates well with the clinical scores of tremor [22]. Then the total peak power in all axes of the accelerometer and gyroscope signals is the spectral power distribution of all axes’ data around the valid dominant frequency with the PSD method. The peak power in all axes after normalization is regarded as the amplitude of tremor.

**Figure 3:**
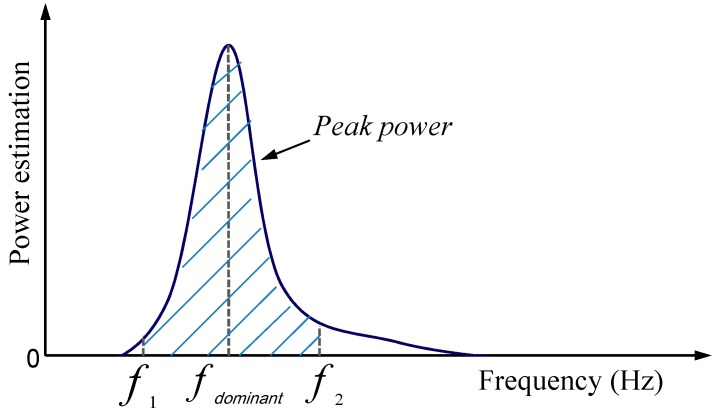
Power spectral density (PSD) estimation of ten-second inertial sensor signals. *f_dominant_* represents the dominant frequency of the signals.

For PSD estimation, peak power means the power estimation around the dominant frequency in the power spectrum of a sensor signal. The peak power in this study is the power estimation around the dominant frequency with ±0.3 Hz length in the single sided power spectrum of ten-second sensor signals [21]. Figure 3 shows the system diagram of peak power, which means the power estimation around the dominant frequency in the sensor signals’ power spectrum.

Equation (1) shows the calculation of the peak power of IMU signals:

(1) $$Peak\;power=\int_{f_{1}}^{f_{2}}\frac{FFT^{*}(signals)\times FFT(signals)}{N^{2}}df$$

where * denotes the complex conjugate and N is the received sample points in the sensor signals.

The frequency resolution is *f_sample rate_*/*N*, while *f_sample rate_* is the sampling frequency.

Therefore, the power spectrum is converted into a single-side power spectrum. The power spectrum magnitude (peak power) has units of the input signal unit-RMS squared. The power estimation units of IMU signals are (°/s)^2^/Hz and g^2^/Hz, respectively. Here, g is equal to m/s^2^.

### 3.5. Tremor State Detection

The occurrence of tremor in a patient also depends on many factors, especially motor fluctuations and the patient’s mental and physical status. Tremor may sometimes disappear even in a patient with severe tremor. Therefore, it is important to quantify tremor severity during the stable tremor state. The tremor state is classified into two types in this study: valid state and invalid state. The sensor signals in an invalid state will be discarded because the patient’s tremor signal cannot correctly be measured.

After a ten-second tremor-assessment task, the tremor signals need to be checked. Valid state means the sensor signal is stable in both the time domain and frequency domain. The judgments of valid state are listed as follows:

1. Frequency domain: In the frequency domain of the ten-second signals, the proportion of peak power to the whole power estimation should be bigger than 85%.
2. Time domain: In the time domain, the SD (standard deviation) value of ten-second angular velocity ranges (peak-to-peak values of all axes of the gyroscope) should be bigger than 70% of the mean gyroscope signal ranges.

For the valid state detection, only the gyroscope signal was utilized in present study. Parameters for the valid state detection both in the time domain and frequency domain are:

(2) $$V_{t}=1-\frac{SD\left(G_{P-P}\right)}{Mean\left(G_{P-P}\right)}$$

(3) $$V_{f}=\frac{P_{peak}}{\sum P_{i}}$$

where *G_p-p_* is the matrix of peak-to-peak values of the combined triple-axis gyroscope signals during a ten-second tremor task, Mean represents the mean value, *P_peak_* is the power estimation around the dominant tremor frequency (±0.3 Hz), *P_i_* is the power estimation of a frequency point in the PSD estimation, while ∑*P_i_* is the power distribution in the whole frequency domain.

### 3.6. Least-Square-Estimation Model and Signal Flow Diagram

Because accelerometers and gyroscopes are used to measure linear and rotational movement, respectively, for some tremor tasks the accelerometers present a higher correlation, while the gyroscopes perform a higher correlation during other tremor assessment tasks. Thus, a multiple linear regression model is used to fit the clinician ratings (UPDRS tremor score) and the peak powers during each tremor task [29,31,35,36].

Then the linear regression model can be expressed as:

(4) $$R=R_{0}+\ln\left(\sum_{i=1}^{3}b_{i}\cdot PA_{i}+\sum_{i=1}^{3}c_{i}\cdot PG_{i}\right)$$

where *R* is the predicated tremor score; *R*_0_, *b_i_*, and *c_i_* are the regression coefficients. *i* = 1, 2, and 3 denotes *x*, *y*, and *z* axis, respectively; *PA_i_* and *PG_i_* are the single-axis peak power for the triple-axis accelerometer and triple-axis gyroscope, respectively [20]. *R*_0_ and the three scaling factors (*b_i_* and *c_i_*) are different for the three tremors (rest, posture, and action) [37]. These three regression coefficients (scaling factors) were obtained according to Stevens’ power law in psychophysics [38].

**Figure 4:**
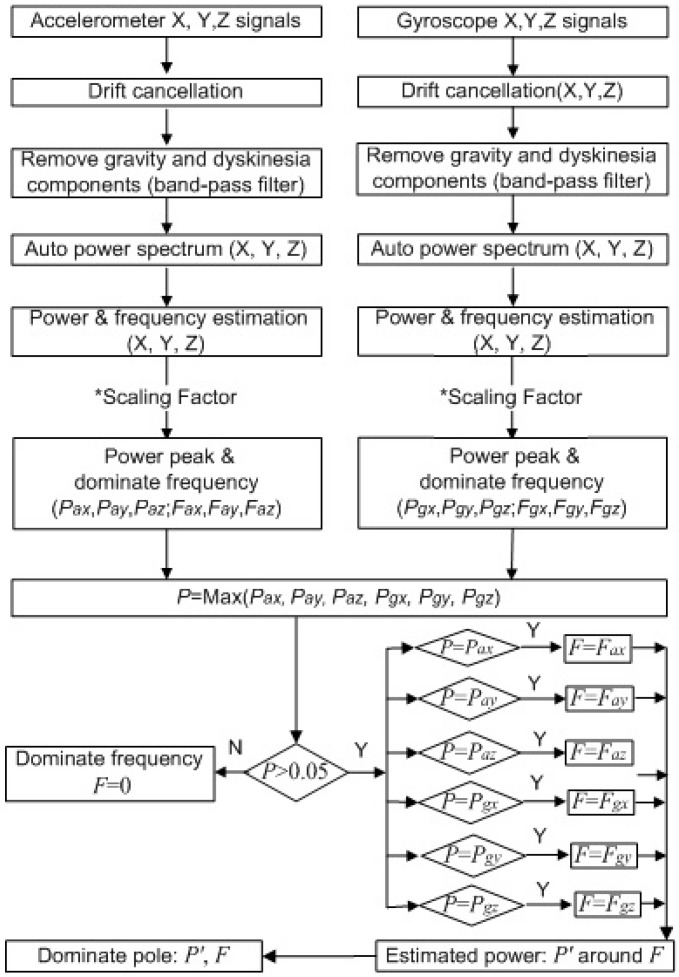
Signal processing for the tremor detection and quantification (six channels).

The flowchart of the signal processing for the tremor quantification in this study is shown in Figure 4. The sensor data are from the glove part, which is based on the IMU board and command module. In the computer part, the sensor data are band-pass filtered from 3.25 to 12 Hz (tremor band) before tremor amplitude quantification, in order to remove gravitational acceleration and dyskinesia components.

The signals in ten seconds are used for the PSD estimation at the end of the ten-second tremor-assessment task. Initially, the signals from the gyroscope and the accelerometer are analyzed separately. The single-sided, scaled, and auto power spectra of the signal of each gyroscope axis and each accelerometer axis, of which length is set to ten seconds, are separately computed to get the four-channel power spectra in real-time. The dominant frequencies and the estimated peak powers of these frequencies are thus obtained. Then, the estimated peak powers are multiplied by scaling factors. The channel with the highest peak power is the dominant channel, and its dominant frequency is the dominant frequency (*F*) of all channels. Other channels are needed to perform PSD estimations again with the dominant frequency, and multiply the peak powers by the scaling factors again. The power estimations in all axes around the same dominant frequency (*F*) are calculated and sum up to *P’*.

Then, the sum of peak powers, which is regarded as the tremor amplitude, and the dominant frequency can be displayed in the recoding list.

At the same time, the power spectrum of every axis in a certain period (three seconds) is calculated continuously. The dominant frequency and peak power can be obtained as soon as the tremor occurs. When the dominant frequency is in the range of 3.5–7.5 Hz and the sum of peak powers is more than a threshold, the tremor is reported.

## 4. Experimental Section

### 4.1. Validation of Analytical Methods

Neurologists judge tremor severity according to its position information, but IMU raw data (acceleration and angular velocity) are used for tremor quantification.

As described in [14], we have compared the tremor assessment systems based on an IMU with an electromagnetic (EM) motion tracking system (NDI Inc., Brewton, AL, Canada), whose RMS accuracy is 0.30° [39]. The EM sensor has small dimensions and was placed on the top of the index finger, together with the IMU board. An experiment for analytical validation of tremor quantification was then carried out. Detailed description is presented in this part.

#### 4.1.1. Hypothesis

In comparison with those from the EM system, the tremor amplitude (peak powers) and dominant frequency of the tremor assessment system should meet the following requirements [40,41]:

- Mean value and standard deviation of the differences between dominant frequencies: *f_md_* < 1.00 ± 0.88 Hz;
- Correlation coefficient of peak powers: *r* > 0.95.

#### 4.1.2. Materials

- NDI (Northern Digital Inc*.,* Waterloo, ON, Canada) Aurora^®^ EM tracking system with a six degree-of-freedom (DOF) sensor;
- Tremor assessment system (including a USB cable and a command module with a sensor board);
- Laptop with the EM system (Aurora Toolbox) application software;
- Laptop with the application software of the tremor assessment system (LabVIEW-based user interface for tremor assessment; MATLAB (Natick, MA, USA) R2012b for data analysis).

#### 4.1.3. Experimental Setup and Method

**Figure 5:**
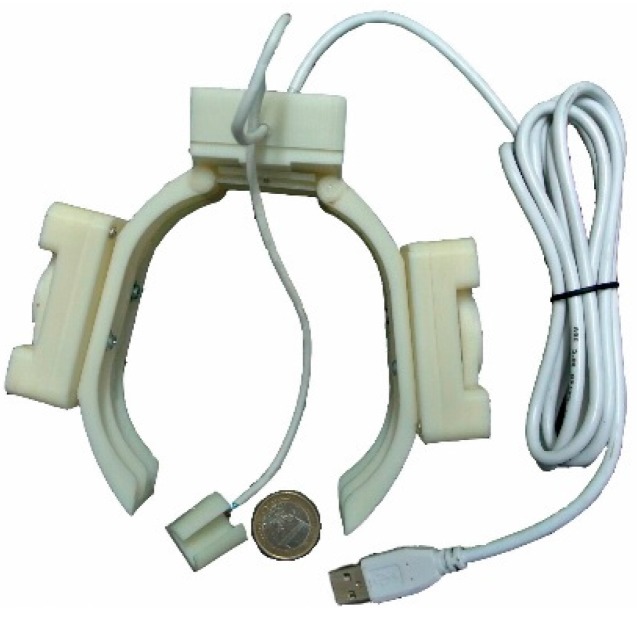
Prototype implementation of the assessment system (glove part) for parkinsonian motor symptoms. In addition to tremor assessment, this modified version of the assessment system can be used to assess bradykinesia, dyskinesia, and rigidity.

Figure 5 shows the assessment system for parkinsonian motor symptoms.

Nine healthy subjects performed postural simulative tremor tasks with different amplitudes, which were recorded by both the IMU-based and EM-based tracking systems. Dominant frequencies and amplitudes between the two systems were compared.

The dominant tremor frequencies calculated by the IMU-based tremor assessment system and EM system were *f_tb_* and *f_em,_* respectively, which were calculated by FFT method. The difference of their dominant frequencies in a single tremor task was then represented by:

(5) $$f_{d}=\left|f_{tb}-f_{e\text{m}}\right|$$

Therefore, *f_md_* (mean and standard deviation of dominant frequency differences) can be calculated using the values of *f_d_* (frequency differences) during all imitated tremor assessment tasks.

The tremor amplitude (peak powers based on the IMU signals) calculated by the tremor assessment system was *R*, while the tremor amplitude (peak power based on the position data) estimated by the EM system was E. Then the correlation coefficient between these two parameters was *r*.

#### 4.1.4. Results

The peak power (amplitude) of the tremor assessment system was calculated with both gyroscope and accelerometer signals using the PSD method, while the peak power of EM signals was calculated only from the position signals of the subject’s finger.

For the postural tremor task, the differences of dominant frequencies between the two systems were small in all situations (*f_md_* = 0.12 ± 0.14 Hz). The maximum difference was 0.57 Hz. Therefore, *f_md_* was smaller than 1.00 ± 0.88 Hz.

The correlation coefficient (*r*) of dominant frequency between the tremor assessment system and EM system was 0.996.

For stable movements (*i.e*., means consistent frequency and range) performed by the subjects, the correlation between the two peak powers of the tremor assessment system and the EM tracking system was approximately linear. The correlation coefficient between these two systems (*r* = 0.97, *p* < 0.001) was larger than 0.95.

Results also show that inconsistent tremor actions resulted in smaller tremor amplitude when using the PSD method. On the other hand, tremor amplitude increased due to that fact that some sampling points were missing.

### 4.2. Clinical Experiments of Tremor Quantification

Clinical experiments of patients were carried out employing the tremor assessment system.

#### 4.2.1. Hypothesis

The tremor amplitude calculated by the tremor assessment system was *R*, while the tremor amplitude judged by the neurologist was *D*.

The tremor amplitude correlation between the tremor assessment system and the clinical ratings should meet the requirement [21]:

- Correlation coefficient: *r* > 0.84.

#### 4.2.2. Experiment Setup

A total of seven patients with Parkinsonian tremor were tested by the tremor assessment system. Their tremor severities (UPDRS tremor scores: *D*) ranged from 1 to 3. The measurements of these patients’ tremors were performed after their medication had been stopped for more than 24 h.

Rest, postural, and action tremor assessment tasks were sequentially performed. However, for each patient, only the side with the more severe tremor was assessed. Each tremor assessment task lasted for ten seconds.

Time-frequency analyses and statistical analyses were carried out based on the IMU signals [42]. The valid state detection algorithm was executed for each assessment task.

#### 4.2.3. Results

**Figure 6:**
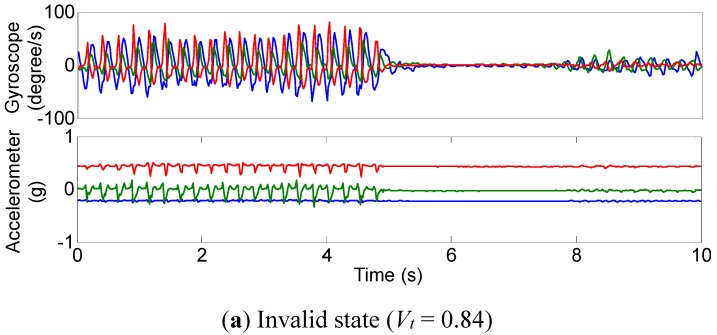

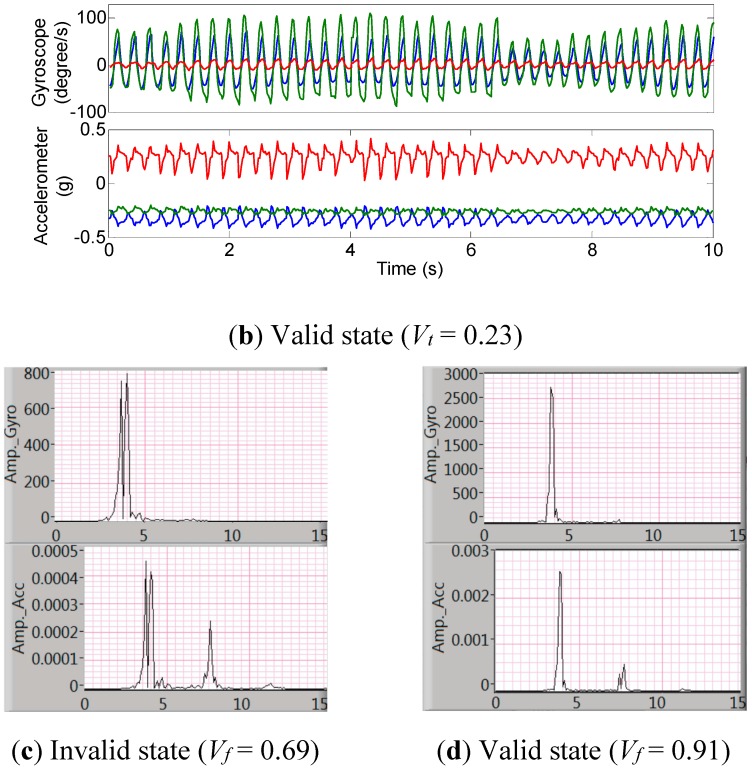
Tremor state for a patient with parkinsonian tremor (UPDRS score *D* = 1): (**a**,**b**): The three-axis angular velocity and three-axis acceleration waveforms of rest tremor; (**c**,**d**): The three-axis combined power spectra of angular velocity signals (**bottom chart**) and acceleration signals (**upper chart**).

Figure 6 shows the IMU signals of the tremor assessment system and their power spectra for a patient with slight parkinsonian tremor (*D* = 1). In Figure 6a, the tremor was disappeared suddenly. However, the tremor amplitude fluctuated only on a modest scale in Figure 6b.

The validation results of tremor quantification and valid state detection algorithms demonstrate that the tremor amplitude calculated using PSD method is based on the premises that the tremor range is stable and its peak power is sharp.

When performing the signal processing algorithms, the rest and postural tremor tasks were assessed as either a valid state or an invalid state for PSD estimation. The amplitude of the action tremor is not included in the present results.

Therefore, the signals in Figure 6b could be used to calculate the tremor amplitude. The assessment tasks whose signals had invalid parameters (*V_t_* > 0.7 or *V_f_* > 0.8) were discarded.

Figure 6b and d show a valid tremor task (*D* = 1), in the time domain and frequency domain, respectively [35].

For the action tremor assessment, it was difficult to extract the tremor from voluntary movement because there were multiple frequency components across its power spectrum [39]. A better signal filter should be employed in the future [43].

The peak power from all IMU outputs (*R*) had the greatest correlation with the clinical scores (*r* = 0.98).

In addition to the linear regression model for tremor quantification, which involves both gyroscope and the accelerometer outputs, the tremor amplitude calculations of the gyroscope and the accelerometer were analyzed independently. Table 1 indicates the calculated parameters together with the clinical scores, which resulted from a neurologist’s judgments using UPDRS ratings. These parameters were the mean values from valid postural and rest tremor assessment tasks.

Here *acc.* means accelerometer; *gyro.* represents gyroscope; *RMS* means root-mean-square; ln denotes the natural logarithm; *R* represents the predicated tremor score from the tremor assessment system; and *r* denotes the correlation coefficient between the relative parameters calculated by the IMU signals and clinical scores judged by the neurologist. For ln(*gyro.power*) and *R*, the negative values are can be regarded as zero because these values are too small.

Table 1 shows that the gyroscope signal had a stronger correlation (*r* = 0.93) with the clinical scores than the accelerometer signal (*r* = 0.88 and 0.91 for *peak powers* and *RMS*, respectively).

Table 1 also shows that the proportion of rotational parts and translation parts of a tremor (*i.e.*, ln(*gyro. power*) *versus* ln(*acc. power*)) varies from patient to patient.

**Table 1:** Results for rest and postural tremor assessments

Subject	ln(*acc. power*)	ln(*gyro.power*)	ln(*acc. RMS*)	ln(*gyro. RMS*)	*R*	Clinical Score(*D*)
Patient 1	−8.52	0 (−6.27)	−2.95	0.30	0 (−5.41)	0.0
Patient 2	−7.42	0.78	−2.42	1.93	0 (−4.01)	0.5
Patient 3	−3.06	7.05	−0.92	3.92	1.27	1.0
Patient 4	−4.10	7.37	−1.40	4.14	1.36	1.5
Patient 5	−2.74	7.87	−0.58	4.31	1.99	2.0
Patient 6	−2.12	7.55	−0.49	4.22	2.06	2.0
Patient 7	−1.95	11.21	−0.22	5.61	2.81	3.0
*r*	0.88	0.93	0.91	0.93	0.98	/
*α* (2-tailed)	0.008	< 0.01	0.004	0.002	<0.01	/

The difference between dominant frequencies from gyroscope and accelerometer were small (< 0.2 Hz). For all the measured patients, the dominant tremor frequency was constant or had only small fluctuations, even if the tremor amplitude changed during ten-second task period.

The results indicate that the tremor frequency was stable for all patients; however, the tremor amplitude fluctuated continuously. The quantitative assessment of tremor, with an IMU and employing adaptive algorithms, provided an objective rating to classify rest or postural tremor severity even on a small scale.

## 5. Conclusions/Outlook

In this study, the features of parkinsonian tremor and its quantitative assessment methods have been presented. A time-frequency signal analysis algorithm for tremor state detection was adopted. After the ten-second tremor-assessment task, the task state is judged, form both frequency and time domains. The task with an invalid state will be discarded, while the signal with a valid state will be further processed via a least-square-estimation model. The peak powers of all axes of accelerations and angular velocities are used to the linear regression analysis. The regression coefficients are different for different tremor types.

A better cognition of the analytical methods in the system based on an IMU was achieved with the validation results. In comparative experiments with an EM tracking system, we showed that the raw data of an IMU correlated well with the severity of imitated tremor tasks (*r* = 0.996). Results also show that inconsistent tremor actions resulted in smaller tremor amplitude when using the PSD method. On the other hand, tremor amplitude increased due to that fact that some sampling points were missing.

Next, the clinical experiments were performed. The clinical experiment showed the assessed severity correlated well with the judgments of a neurologist (*r* = 0.98). In addition, the dominant tremor frequency was constant or had only small fluctuations even if the tremor amplitude changed during the ten-second task period.

This study provides the necessary science and engineering to guide future signal processing methods. Then future works should involve following points:

1. The relation between the peak power of IMU signals and the consistency of IMU signals in PD patients should be studied [43,44].
2. The repeatability of tremor amplitude with the same patient at different times should be investigated further [45].
3. The regression coefficients in Equation (5) are different according to tasks and tremor types. More clinical experiments are needed to modify the scale factors and coefficients in the tremor amplitude calculation. In addition, essential tremor and other types of pathological tremors need to be quantitatively assessed.
4. Together with the assessments of bradykinesia, rigidity, and the side effects of medication or surgical treatments such as dyskinesia, paresthesia, and other non-motor symptoms, more parameters should be evaluated in a single system for the overall assessment of parkinsonian symptoms [16,46].

In addition, there are two main trends in the development of MEMS motion sensors: more sensors embedded in a single chip and more accurate sensor fusion algorithms. These two points make it easier for the future system to detect the hand tremor with even smaller dimensions and greater accuracy. For example, a three-axis compass can remove the yaw drift of the IMU fusion output. Thus, more accurate orientation values, even location values, can be obtained. The new MEMS technology and motion-tracking algorithms will improve the performance of the tremor assessment system.
